# Heterogeneous breast phantom for computed tomography and magnetic resonance imaging

**DOI:** 10.1371/journal.pone.0284531

**Published:** 2023-04-13

**Authors:** Gameel Saleh, Ashraf Abuelhaija, Budour Alfaris, Aljohara Aljabr, Maryam Zainalabedin, M. H. A. Mhareb, Maryam Alhashim, Salma Alenezi

**Affiliations:** 1 Department of Biomedical Engineering, College of Engineering, Imam Abdulrahman Bin Faisal University, Dammam, Saudi Arabia; 2 Department of Electrical Engineering, Faculty of Engineering and Technology, Applied Science Private University, Amman, Jordan; 3 Department of Physics, College of Science, Imam Abdulrahman Bin Faisal University, Dammam, Saudi Arabia; 4 King Fahad Specialist Hospital (KFSH), Dammam, Saudi Arabia; Tsinghua University, CHINA

## Abstract

In this article, a heterogeneous multimodal anthropomorphic breast phantom with carcinoma is introduced to meet the response of the natural breast tissue when imaged using ionizing and non-ionizing machines. The skin, adipose, fibroglandular, pectoral muscle, and carcinoma tissue were mimicked. A T1-weighted breast magnetic resonance image with BI-RADS I tissue segmentation was used for molds creation. The tissue-mimicking materials (TMMs) were tailored in terms of their elemental composition weight fractions and their response to ionization radiation parameters. These are the mass attenuation coefficient (MAC), electron density (*n*_*e*_) and effective atomic number (*Z*_*eff*_). The behaviour of the TMMs, when exposed to a wide range of ionization radiation energy, was investigated analytically and numerically using X-COM. The achieved results showed an excellent agreement with the corresponding properties of the natural breast elemental compositions as reported by the International Commission on Radiation Units and Measurements (ICRU). The MAC of the TMMs and the ICRU-based breast tissue were found to be consistent. The maximum percentage of error in *n*_*e*_ and *Z*_*eff*_ amounts to only 2.93% and 5.76%, respectively. For non-ionizing imaging, the TMMs were characterized in term of T1 and T2 relaxation times. Using our preclinical MRI unit, the TMMs relaxation times were measured and compared to the natural tissue. The fabricated phantom was validated experimentally using CT, MRI, and Mammographic machines. The achieved images of the TMMs were in alignment with the real tissue in terms of CT HU values and grayscale colors. T1W and T2W images on MRI revealed the expected contrast between TMMs as in natural tissue.

## 1. Introduction

Breast cancer is one of the main causes of mortality, particularly in women. One of the factors that might help in breast cancer detection is the existence of breast phantoms that are highly like the real breast associated with the modality used. Heterogenous breast phantom can contribute to improving the accuracy of breast cancer detection in medical imaging systems, by mimicking the real breast tissues way of interaction while exposed to ionizing and non-ionizing radiation. From a manufacturer’s perspective, it helps standardize image quality and machine stability measurements and testing. In this, technicians can adjust the parameters of their imaging modalities to get the ultimate results and eliminate multiple scanning and extra dose emitted into the patient’s body. If the blood vessels’ model is fabricated to connect different types of contrast agents to the tumor location, it can be used to train the system with several types of pulse sequences. Breast phantoms are categorized into physical and computational models [1]. Most physical phantoms were developed using molding processes or 3D printing with limitations of printed materials and meeting the properties of each imaging technology [1]. Currently, most of the available works focus on designing a phantom for a single consistent imaging modality. Some physical anthropomorphic breast phantoms [2, 3] were designed for Mammography. By matching virtual breast phantoms for mammography projections, a group at Duke University developed a breast phantom. The designed phantom had undesirable air bubbles [2]. Another physical 3D breast phantom was created at the University of Pennsylvania for quality assurance (QA) of 2D and 3D breast ionizing imaging systems. The fabricated phantom consists of 45% dense tissue and is 5 cm deformed in thickness for mammography. The phantom showed an acceptable appearance with the presence of air bubbles [3]. A CT Breast Phantom was designed by segmenting the patient’s breast image into both adipose and fibroglandular tissue and made from polyethylene [4]. A thermoplastic mold was placed as the outer layer of the skin to mimic the different tissue. Fibroglandular tissue was made by filling air gaps with water, while polyethylene and paraffin wax were used to mimic adipose tissue. Moreover, calcium carbonate particles were used to represent the macro-calcification [4]. Another breast phantom of CT gel-like material was developed using in credentialing of permanent breast seed implant brachytherapy [5].

A simple design of a phantom that mimics the adipose tissue, normal parenchyma and lesions was presented for MRI [6]. Another study [7] was carried out to design a breast phantom that can serve as a quality control (QC) to promote the standardization of breast MRI quantitative measurements. Additionally, a study [8] was conducted to create a 3D phantom based on a healthy patient’s data for assessing MRI quantitatively. This study showed a good design of the breast phantom related to the MR characteristics, but lack in considering the mechanical properties.

Few studies have been found for designing a multimodality breast phantom for various imaging modalities [9–13]. A multipurpose gel-based breast phantom with a malignant mass was proposed to function with ultrasound, CT, and MRI [10]. The results of the Hounsfield unit (HU) and signal-difference-to-noise ratio (SDNR) were far from the reference values for adipose and fibroglandular for CT and malignant mass for MRI [10]. A breast phantom for multimodality imaging with tissue-mimicking materials (TMMs) based on 3D printing was introduced for CT and MRI considering the HU and MRI relaxation times [11]. The results showed that the temperature variance between the polyvinyl chloride (PVC) softener mixture and the breast mold could present bubbles that affects the image quality and cannot be eliminated. Additionally, the lack of heterogeneity presented in the tissue reduces the similarity to the real breast tissue [11]. A phantom [12] with TMMs was presented to combine the linear attenuation coefficient, dielectric properties, acoustic properties, and relaxation times. The phantom showed a good match between the measured reference and the physical values. However, because of the fat layer, the solid phantom parts were compression irreversible and insufficient contrast between the tumor and the surrounding tissue was observed [12]. A patent was published on a breast phantom as a biopsy training device, and it comprises skin and fibroglandular tissue with lesions [13]. The numerical results for the ionizing radiation properties of the designed TMMs in this introduced phantom were presented initially in [14].

This article aims to design and develop a heterogeneous anthropomorphic multimodality breast phantom that mimics real breast tissue in terms of anatomy and tissue properties. The phantom includes materials that mimic the skin, fibroglandular, adipose, pectoral muscle, and carcinoma tissue. The ionizing and non-ionizing properties of the chosen materials must be consistent with those properties of the natural breast tissue, as specified by the ICRU. These properties are the effective atomic number (*Z*_*eff*_), electron density (*n*_*e*_) and mass attenuation coefficient (MAC) for ionizing imaging modalities, such as MAMO and CT, as well as the T1 and T2 relaxation times parameters for breast magnetic resonance imaging (bMRI).

## 2. Patient data acquisition and phantom design

Digital Imaging and Communications in Medicine (DICOM) breast T1W images were used in this study. The patient data selection was built on BIRADS which was established by the American College of Radiology (ACR). The score for the chosen image was I (normal breast) with dense fibroglandular tissue to be inserted with a malignant lesion. An Institutional Review Board (IRB) approval was obtained from King Fahad Specialist Hospital (KFSH) in Saudi Arabia, Dammam (RAD0319), to use the patient data. The IRB waived the requirement for informed consent. All participants in this article provided informed written consent.

### 2.1. Image segmentation

**Fig 1:**
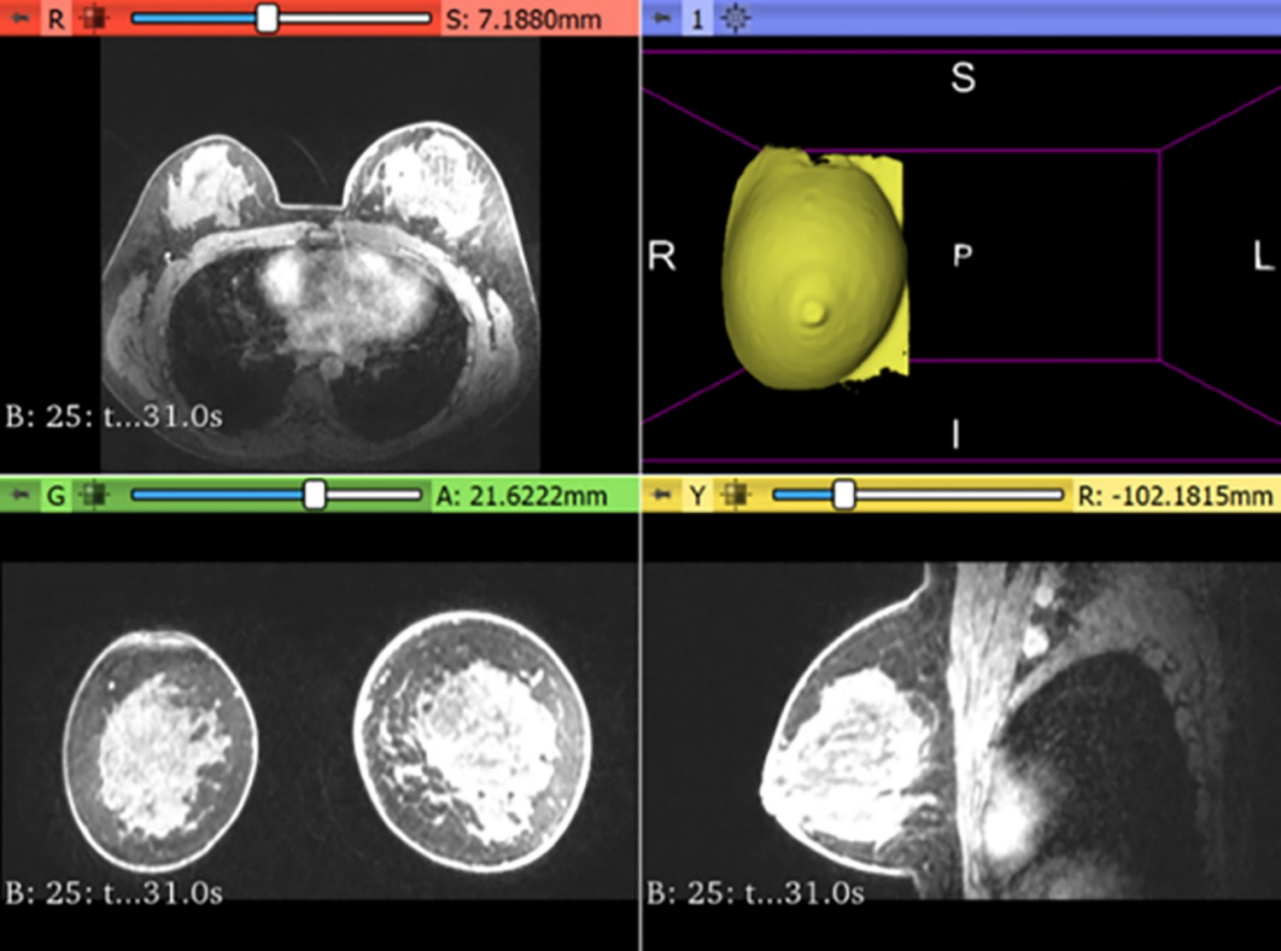
Real patient DICOM images with the segmented skin tissue model from the front view, the upper right image.

For breast tissue segmentation, the acquired MR breast images were imported into segmentation 3D slicer software to have a realistic separated geometry for the external shape, skin, fibroglandular tissue, and tumor. To segment out the fibroglandular tissue from the surrounding adipose and to ensure a complete elimination of all the surrounding structures, the patient DICOM images were imported with several slices for internal tissue segmentation. The slices were segmented from different orientations to improve segmentation reliability using the threshold function. Subsequently, the segmented fibroglandular tissue was converted into a 3D model as a Standard Triangle Language (STL) file, for more processing. The segmentation of the left breast skin from the front view is shown in Fig 1. This part simulates the external shape with realistic deformities, which is needed for mold fabrication. The same steps are repeated for the fibroglandular tissue.

### 2.2. 3D mold processing

**Fig 2:**
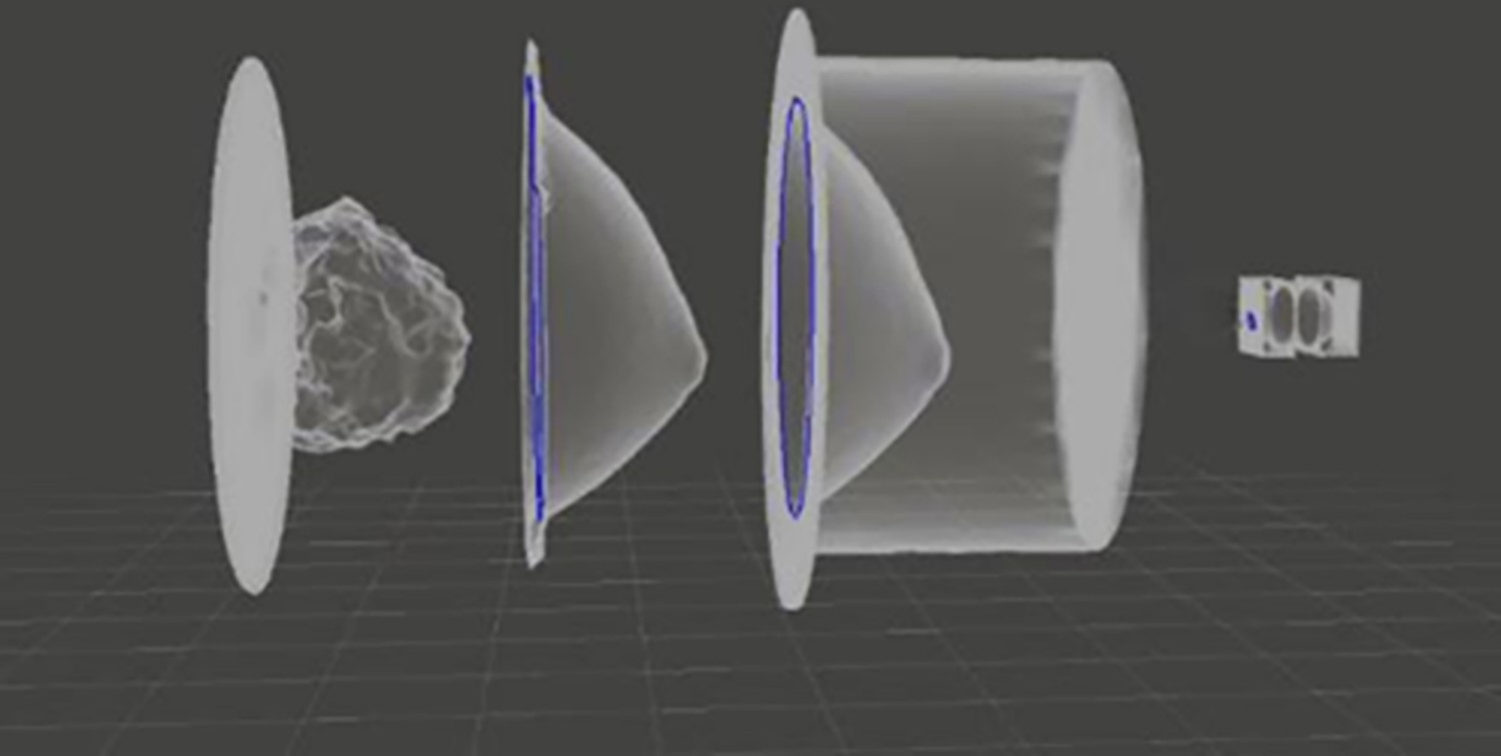
3D phantom design molds. Starting from the left, fibroglandular tissue mold, inner skin layer mold which should assist in creating the skin structure inside the breast, outer skin layer mold which should assist in creating the skin structure outside the breast, tumor mold.

Mesh Mixer software was used for further editing the STL files of the segmented breast images of skin, and fibroglandular tissue as well as the breast’s external shape. Negative and positive molds were created from the segmented skin layer to create a single flask for the skin and adipose tissue. The segmented fibroglandular was modified to create a base for handling purposes. A tumor mold of 2 cm diameter was used to create a carcinoma model. The skin was converted into a negative mold to represent the outer shape of the phantom, with a 3 mm thickness being added at the rounded corners to create a thickness while depressing the mold into the poured skin TMM. Further, the fibroglandular STL file was edited to have an applicable adipose to fibroglandular interface surface for printing reasons. The whole breast phantom molds are shown in Fig 2.

### 2.3. 3D mold printing

**Fig 3:**
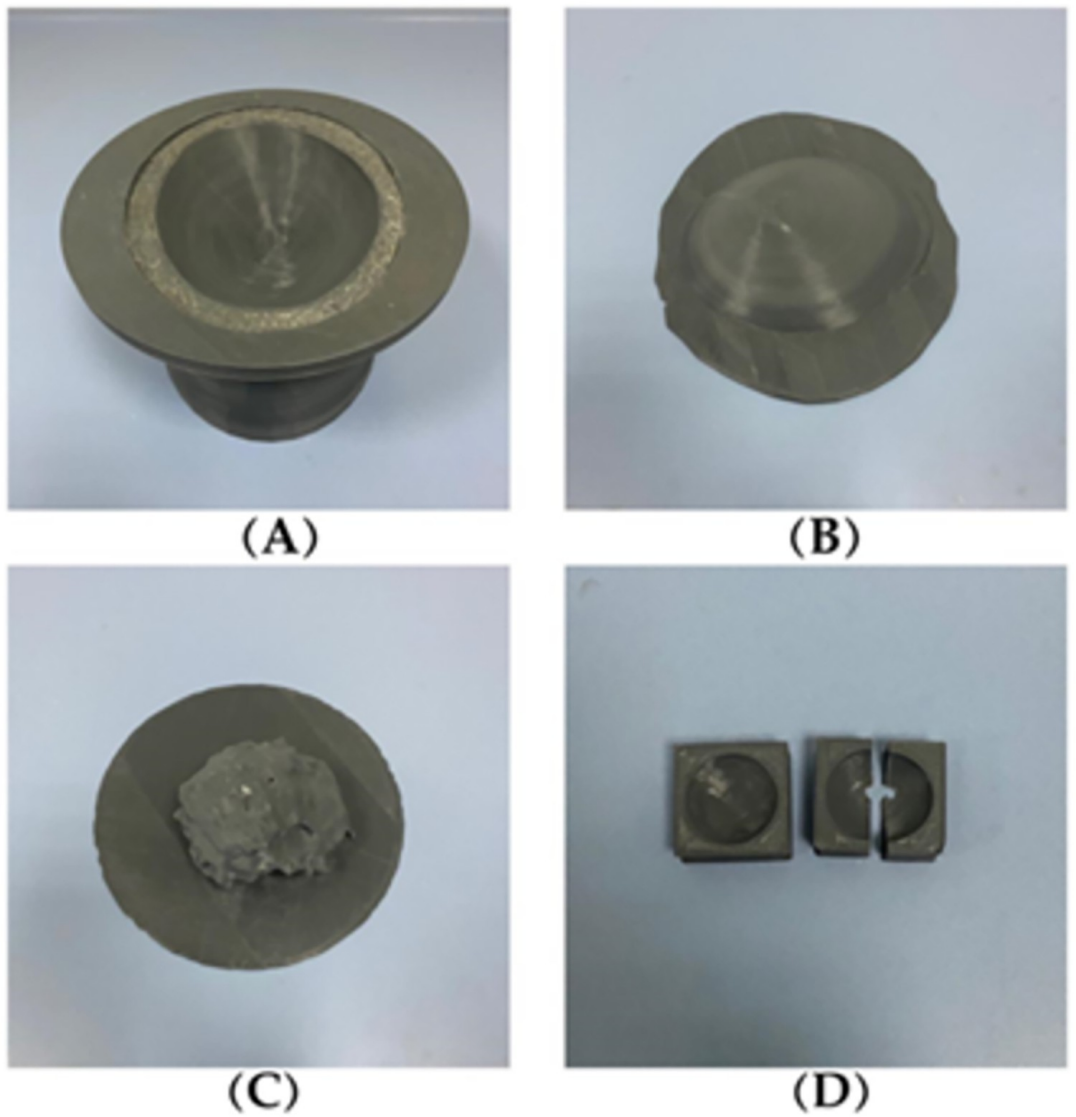
The 3D molds: (A) outer skin, (B) inner skin, (C) fibroglandular, and (D) carcinoma mold.

The phantom molds were extracted from the segmented and processed patient MR breast image data. Four molds were printed that represent the outer breast skin layer, the inner skin layer, the fibroglandular, and the mold for the tumor with a diameter of 2 cm, as shown in Fig 3. The molds of the external breast shape, skin, and fibroglandular were printed using acrylonitrile butadiene styrene (ABS) plastic to have a highly realistic distribution of the interior structure specifically for the fibroglandular mold [15]. The main advantage of ABS material in this application is that its glass transition temperature is around 105°C, which is high enough to withstand the temperature of the mixture. Furthermore, it is highly resistant to physical effects and chemical corrosion, which provides it with the characteristic that it could be used under different environmental conditions [16]. The 3D molds segmented from the patient images were printed using PRUSA 3D printers.

## 3. Tissue mimicking materials characterization

The chosen tissue-mimicking materials (TMMs) were characterized for ionizing radiation modalities such as CT and mammography and non-ionizing modalities such as MRI. The reason behind using the proposed phantom TMMs were based on the real breast tissues elemental compositions weight fractions found from ICRU reports. To use the proposed phantom with ionizing modalities, the TMMs were evaluated using three important parameters, namely mass attenuation coefficient (MAC), electron density (***n***_***e***_) and effective atomic number (***Z***_***eff***_). The weight fractions of the elemental compositions of the tissue were found mathematically from their chemical components, masses, chemical formulae, and molecular weights. The MAC was found numerically using the National Institute of Standards and Technology (NIST) XCOM for each tissue from the weight fractions. The energies were specified according to the range used in each imaging modality, mammography, and CT which is from 10 keV to 150 keV, as clinically used. From the mass density (***ρ***_***m***_) and atomic composition (***Z***/***A***), the electron density of a material was calculated according to the formula:

$$n_{e}=\rho_{m}.N_{A}.\left(\frac{Z}{A}\right)$$ (1)

and

$$\frac{Z}{A}=\sum_{i}a_{i}.(\frac{Z_{i}}{A_{i}})$$ (2)

where ***N***_**A**_ is Avogadro’s number and ***a***_***i***_ is the fraction by weight of the ***i***^***th***^ element of atomic number ***Z***_***i***_ and atomic weight ***A***_***i***_. The mass density for fibroglandular tissue was 1.02 g/cm3 [17], 0.95 g/cm3 for the adipose tissue, 1.09 g/cm3 for the skin layer and 1.04 g/cm3 for the pectoral muscle [18]. For the carcinoma, the mass density was calculated from the fabricated model, and it was found to be 0.44 g/cm^3^. ***Z***_***eff***_ was obtained from:

$$Z_{eff}={\left(\sum_{\begin{array}{c} alltissue\\ components(n) \end{array}}a_{n}Z_{n}^{2.94}\right)}^{\frac{1}{2.94}}$$ (3)

where ***a***_***n***_ denotes the fractional contribution of each element to the total number of electrons in the mixture. The calculated weight fractions of the developed tissue mimicking materials were compared to the natural breast tissue found from ICRU. For the use of the proposed phantom in magnetic resonance imaging, the TMMs were characterized by their intrinsic relaxation times T1 and T2.

### 3.1. Skin, fibroglandular, adipose tissue, pectoral muscle, and carcinoma TMMS

The proposed components for the TMMs in Table 1 were based on choosing specific materials [12, 13, 19–22]. The quantities of all proposed tissue components were tailored to obtain the least error percentage compared to the ICRU breast reference values. This is to provide realism to mimic the representation of natural breast tissue with an improved anthropomorphic nature, as well as to meet the response of the breast characteristics. Deionized water and safflower were introduced to all TMMs to simulate the water and oil concentrations in the tissue, respectively. The carcinoma TMM does not contain safflower oil since it simulates a watery malignant tumor. Additionally, Sodium chloride, Aluminum oxide, and Potassium chloride were used as scattering particles when the tissue is introduced to the X-rays, and to affect the attenuation with a linear dependence as a function of frequency.

**Table 1:** The components, in grams, of the proposed tissue mimicking materials (TMMs).

Layer	Component	Weight (g)
Skin	Deionized water	360
	X-100 surfactant	20
	PVA	40
	Benzalkonium chloride	2
	Sugar	240
	Safflower oil	80
Adipose Tissue	NaCl	0.5
	Deionized water	27.5
	X-100 surfactant	25
	Safflower oil	66
	Olive oil	12.5
	Beeswax	100
	Agar	1.75
	KCl	1
Fibroglandular Tissue	SiC	1
	Deionized water	165.95
	X-100 surfactant	10
	Safflower oil	42.5
	Glycerol	32.5
	Agar	6.75
	Aluminum Oxide	3.75
	KCl	1
	Benzalkonium chloride	1.25
Carcinoma	NaCl	1.75
	Agar	8.75
	Sugar	55
	Deionized water	175
	Benzalkonium chloride	0.875
	KCl	0.475
Pectoral muscles Tissue	Agar	12
	Sugar	180
	Deionized water	436.5
	Benzalkonium chloride	2.5
	X-100 surfactant	10
	Safflower oil	40

### 3.2. Tissue mimicking materials fabrication

**Fig 4:**
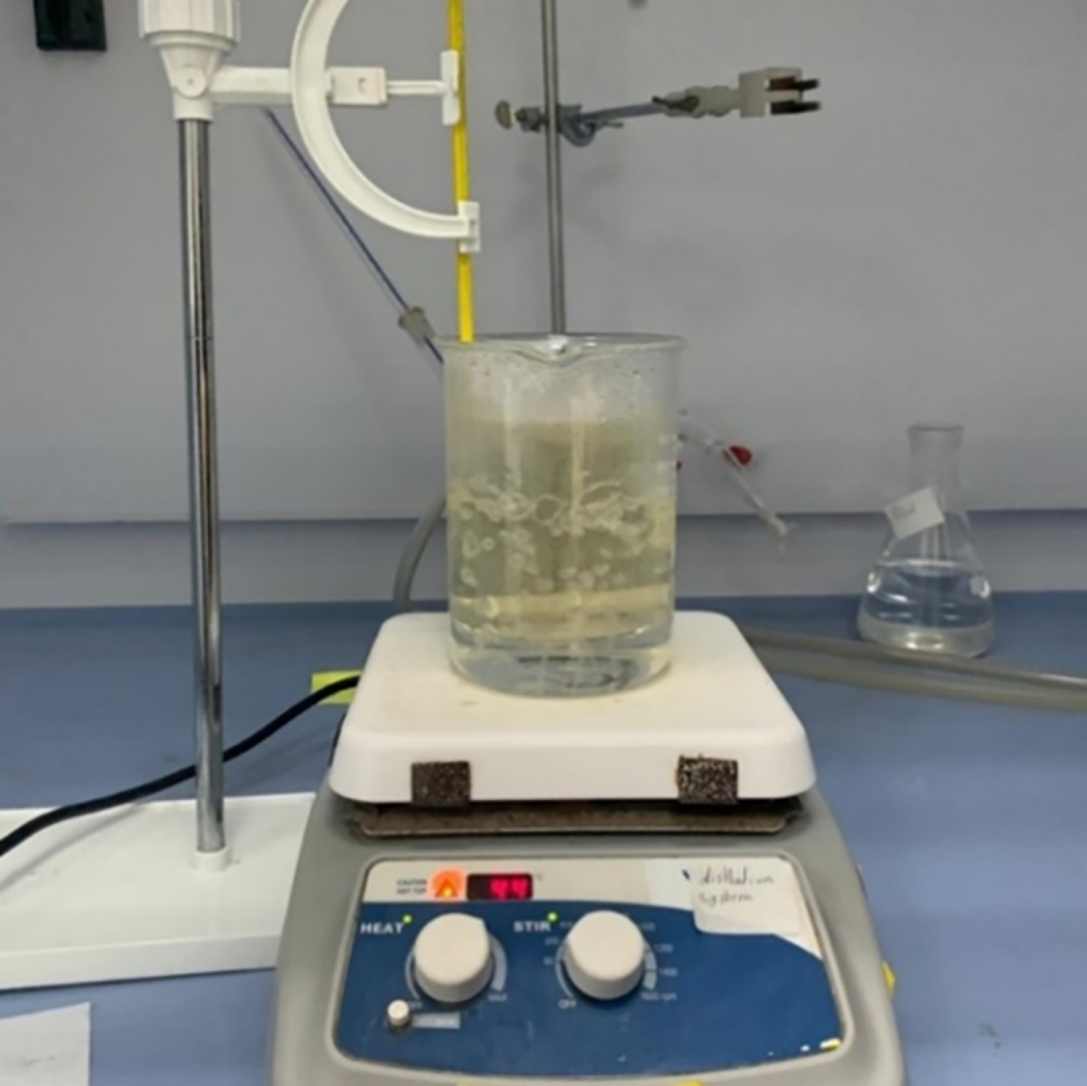
Skin tissue under fabrication process.

This section includes the fabrication process for the five TMMs. Regarding the skin TMM which is a combination of water and fat mixture, the following steps were made with the specified quantities in Table 1:

- The safflower oil was poured into a glass beaker along with the specified quantity of the surfactant, followed by mixing the two liquids with a blender till thick white consistency appeared.
- Another glass beaker was used for the water mixture, including the specified quantity for both deionized water and benzalkonium chloride, followed by adding to it the specified amounts of PVA and sugar after they were thoroughly mixed.
- The water mixture beaker was set into a water bath with a temperature set to 94° C and mixed with a magnetic stirrer at 200 rpm for 5 minutes and then at 100 rpm for 55 minutes, as shown in Fig 4.
- After boiling the water for half an hour, the fat mixture was placed in a water bath until the temperature exceeded 80°C.
- After one hour, the fat mixture was added to the water mixture completing the mixing with the magnetic stirrer for 10 minutes.
- Finally, the mixture was removed from the water bath and allowed to cool until the temperature reached 55°C and was ready to be poured.

During skin molds fabrication, the mechanical properties such as handling the whole fabricated tissue inside it without any corrosion cannot be reached with the real skin thickness. The fabricated skin thickness has been increased for this reason.

The fibroglandular tissue was based on agar-based TMM and fat mixture, and the following steps were made with the specified quantities in Table 1:

- The powder mixture of agar, silicon carbide, aluminum oxide, and potassium chloride was weighted, put in a glass beaker, and thoroughly mixed.
- The liquid mixture of deionized water, glycol, and Benzalkonium Chloride was weighted, put in a glass beaker, and mixed until the benzalkonium chloride completely dissolves in the solution.
- The powder mixture was added to the liquid mixture and of the mixed unit, the water bath reached the desired temperature.
- Steps III to VI in skin production are repeated.

**Fig 5:**
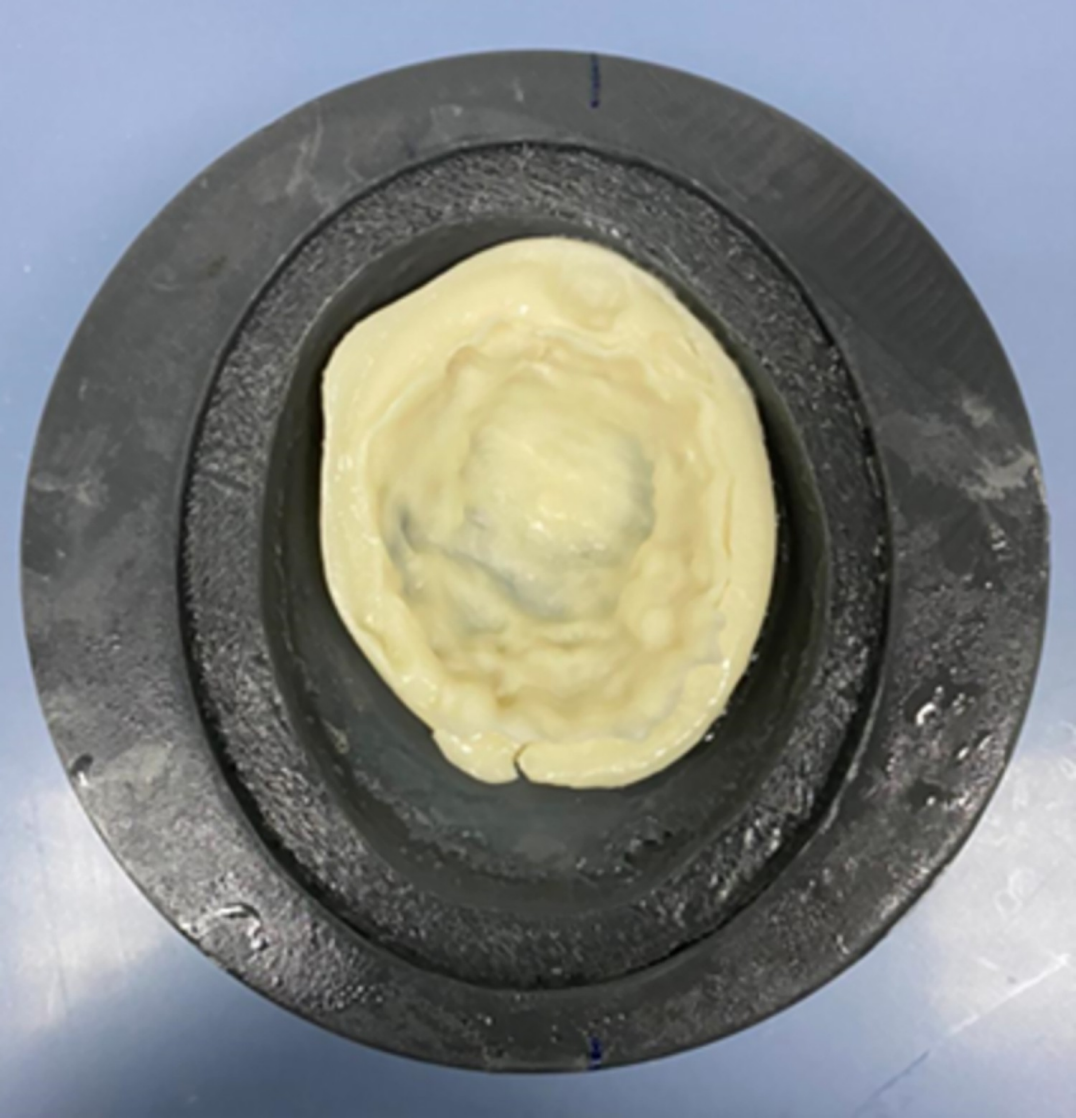
Adipose tissue after pouring into the mold.

For the adipose TMM, a Beeswax mixture and a water mixture were made and added together as the following steps, and with the specified quantities in Table 1:

- The beeswax mixture was first prepared by measuring the beeswax and adding it to a beaker; then the olive oil and safflower oil were measured and added too.
- Another beaker was used for the water mixture. To begin with, the deionized water was measured and added then the specified measurements of the NaCl, KCL and agar were added into the beaker and mixed with the deionized water.
- The beaker with the beeswax mixture was set into a water bath with a temperature of 94°C until the beeswax was melted. Then it was mixed, and the pure surfactant was added to the mixture.
- The beaker with the water mixture was placed in a water bath and mixed with a magnetic stirrer until the temperature reached more than 80° C.
- Then the water mixture was added to the beeswax mixture and have been mixed with the magnetic stirrer for 5 min.
- Finally, the mixture was removed from the water bath, and it was allowed to cool until it reached 55°C with continuous mixing, then it was ready to be poured.
- After pouring it and pressing the adipose mixture with the mold until it was solidified, as shown in Fig 5.

**Fig 6:**
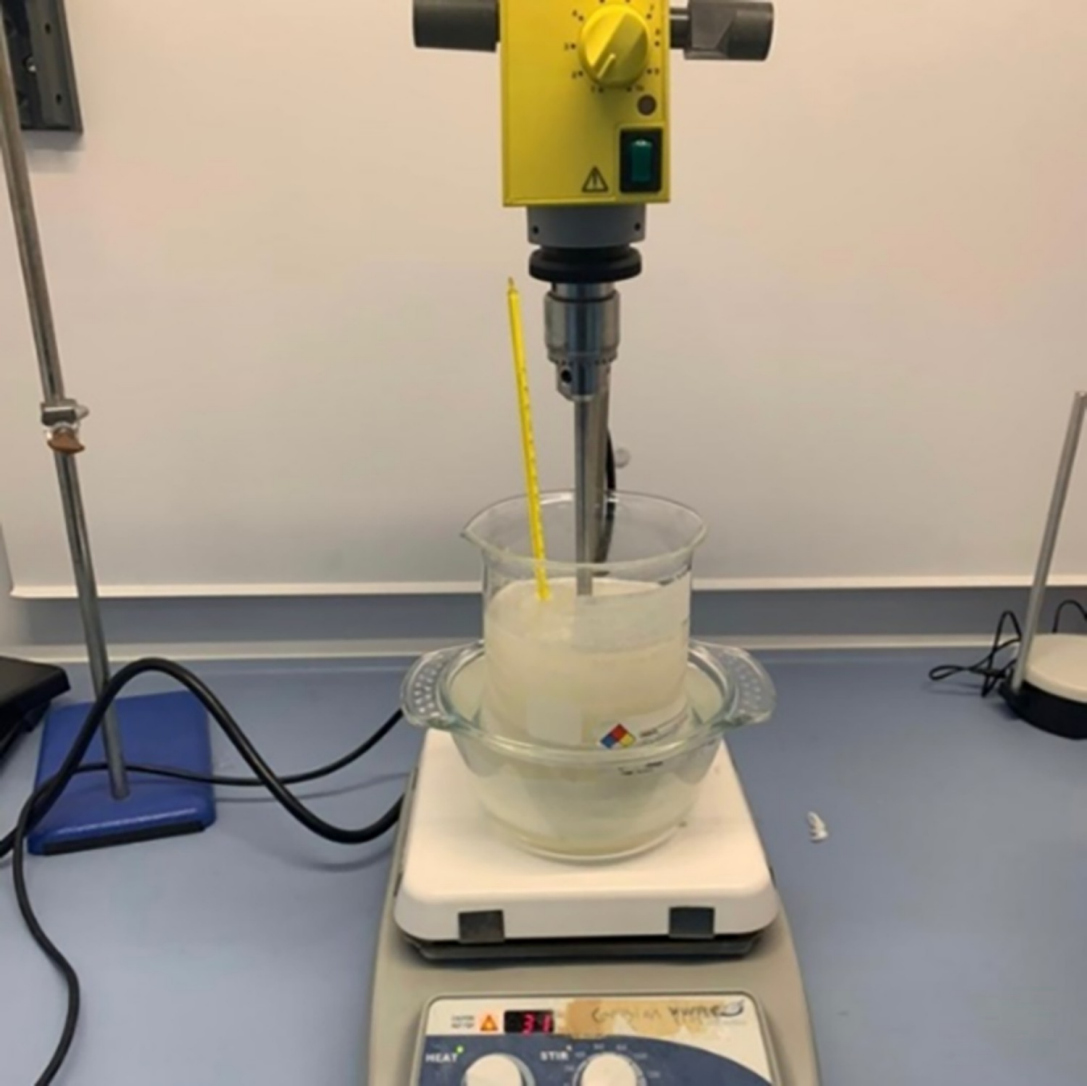
The pectoral muscle under the fabrication process.

For pectoral muscles, a mixture of water and fat was made and mixed according to the specific quantities as in Table 1, and as the following steps:

- A specific amount of agar and sugar were mixed in the beaker.
- Deionized water and Benzalkonium were mixed thoroughly in another beaker.
- Slowly and with continued mixing, the deionized water and Benzalkonium mixture was added to the agar and sugar mixture.
- Steps III to VI in skin production were repeated, as shown in Fig 6.

**Fig 7:**
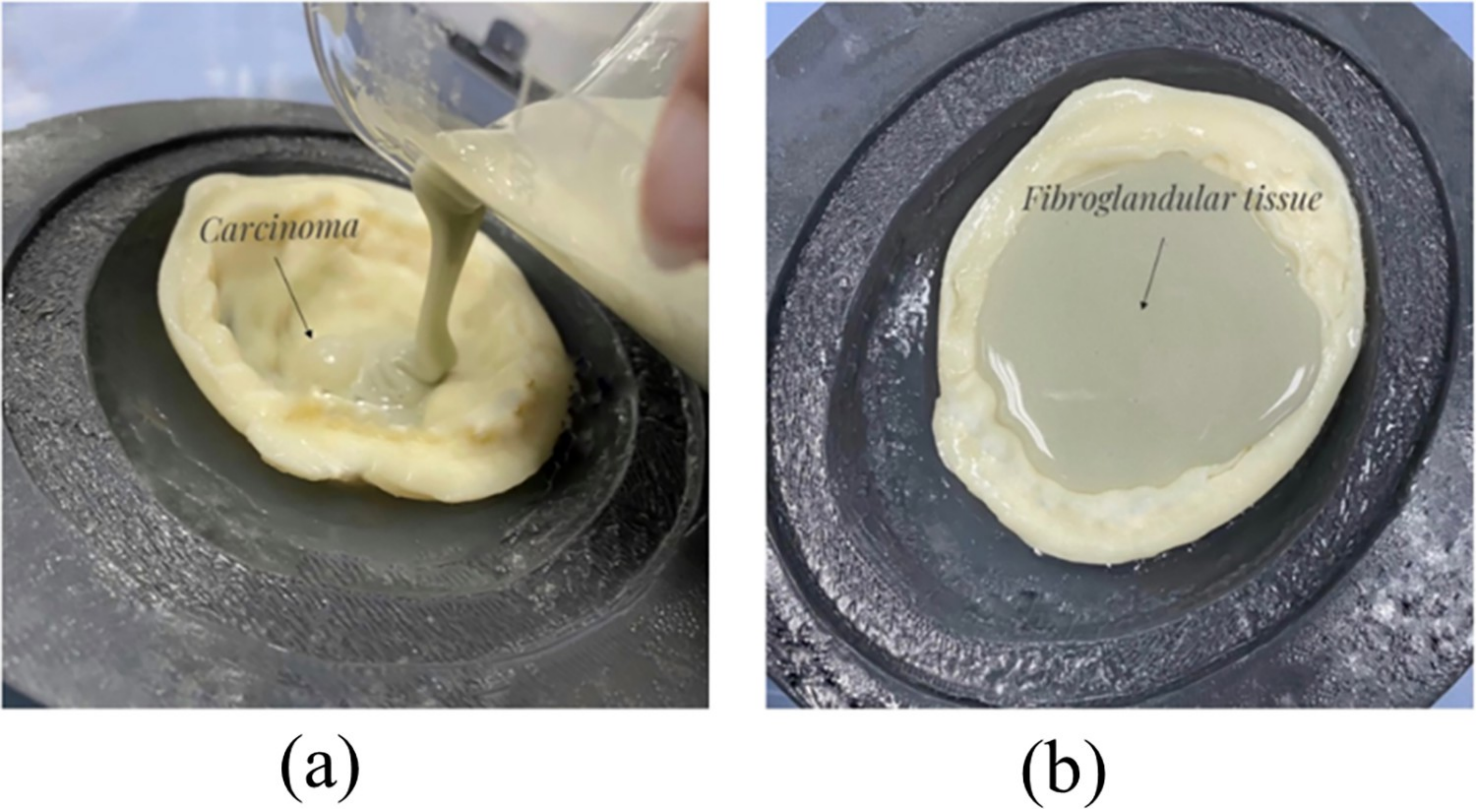
(a) Carcinoma TMM inside the adipose layer. (b) The fibroglandular TMM poured above the carcinoma and adipose layer.

For the carcinoma TMM, agar material as in fibroglandular and pectoral muscle is used with the specified quantities of materials shown in Table 1, and as the following steps:

- The powder mixture of agar, sodium chloride, sugar, and Potassium Chloride was weighed, placed in a glass beaker and mixed thoroughly.
- The liquid mixture of deionized water and Benzalkonium Chloride was weighed, put in a glass beaker and mixed until the Benzalkonium Chloride dissolved in the solution.
- The powder mixture was added to the liquid and mixed until the water bath reached the desired temperature.
- The glass beaker with the mixture was introduced to the water bath when the temperature reached 94°C. The solution inside the water bath was set on the magnetic stirrer with a speed of 200 rpm for 5 minutes and then 100 rpm for 55 minutes. The solution temperature was monitored and kept at 90° C throughout the magnetic stirring process.
- The solution was removed from the water bath and allowed to cool to 45° C with mixing at 55 rpm, then poured into the mold; see Fig 7.

## 4. Breast phantom fabrication

**Fig 8:**
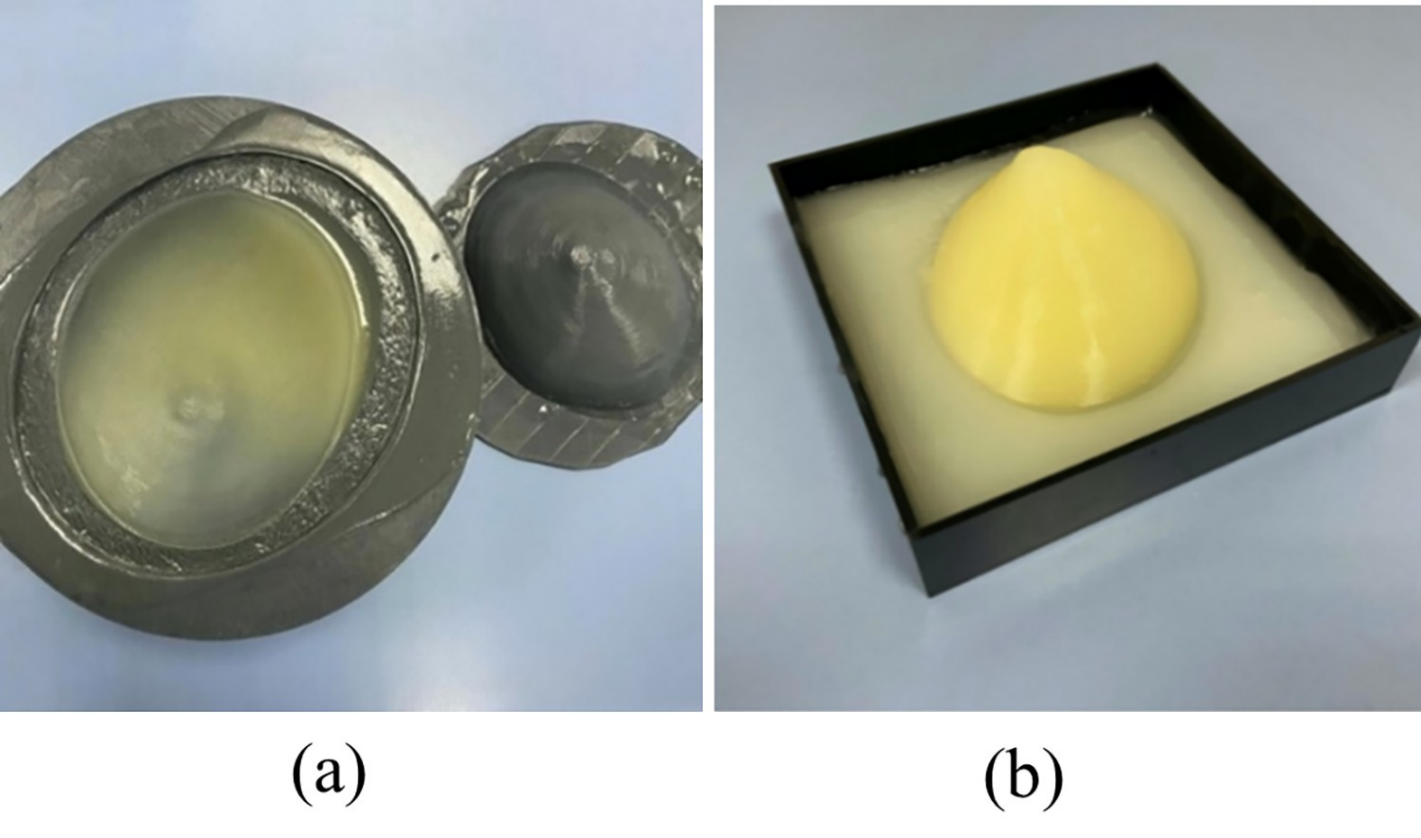
(a) Fabricated phantom skin layer; (b) Fabricated breast phantom after solidification.

The breast phantom is covered with a skin layer and pectoral muscle around it. After the skin layer had solidified in the external skin layer mold as shown in Fig 8(A), the adipose tissue was poured above it and shaped using the fibroglandular interface mold. The carcinoma was placed after adipose solidification, see Fig 7(A). While the fibroglandular tissue was poured above the adipose and carcinoma, see Fig 7(B). The fabricated breast phantom had a highly realistic appearance as shown in Fig 8(B). In addition, the phantom was stored at a temperature of less than 40°F to avoid any drying and deterioration matters.

## 5. Results and discussions

### 5.1. Ionizing properties of the chosen TMMs

For using the introduced phantom for CT and MAMO, the weight fractions of the proposed TMMs elements were calculated for each breast tissue of the skin, fibroglandular, adipose, pectoral muscle, and malignant carcinoma based on the chemical formulae of the chosen materials.

Table 2 presents the calculated weight fractions of the elemental compositions of TMMs comparable to the natural breast tissue found in ICRU reports. The values were close to the natural elemental compositions, especially when focusing on the main tissue elements which are C, H, and O for all tissue.

**Table 2:** Breast phantom elemental compositions weight fractions.

		Na	Cl	C	H	O	Mg	K	N	S	P	Si	Al
Skin	TMMs	0.004821033	0.007692516	0.275323873	0.098487258	0.613573165	-	-	0.000102156	-	-	-	-
	ICRU	0.001	0.003	0.204	0.1	0.645	-	0.001	0.042	0.002	0.001	-	-
Adipose	TMMs	0.000832225	0.004552743	0.689825033	0.12133871	0.17621494	-	0.003605935	-	-	-	0.003630414	-
	ICRU	0.001	0.001	0.598	0.114	0.278	-	-	0.007	0.001	-	-	-
Fibro- glandular	TMMs	-	0.002619926	0.218820618	0.105858707	0.659929689	-	0.002477372	0.000147657	-	-	0.002646044	0.007499987
	ICRU	0.001	0.001	0.332	0.106	0.527	-	-	0.03	0.002	0.001	-	-
Pectoral Muscles	TMMs	-	0.000234935	0.16631	0.096804967	0.73072	-	-	0.005929387	-	-	-	-
	ICRU	0.0008	-	0.123	0.101997	0.720993	0.002	0.0002	0.035	0.005	0.002	-	-
Carcinoma	TMMs	0.002849483	0.005615315	0.116584436	0.098753539	0.776084016	-	0.001031131	0.000113212	-	-	-	-
	ICRU	-	-	0.187626775	0.101419878	0.668356998	-	-	0.042596349	-	-	-	-

Based on the weight fractions in Table 2 and Eqs (1), (2), and (3), the electron density and effective atomic number for both natural breast tissue and the proposed phantom were calculated. We observed low error percentages between natural and mimicked tissue, with a maximum of 5.76% for the effective atomic number, and 2.93% for the electron density, see Table 3. Despite not all the elements were exactly matched the real elements found in the natural breast from ICRU, the proposed materials and quantities produced similar natural breast tissue interactions when exposed to ionizing radiations with energies in the range of 10–150 keV used clinically for breast diagnosis.

**Table 3:** Skin, fibroglandular, adipose, pectoral muscles, and carcinoma electron density and *Z*_*eff*_.

Tissue		Electron density	Error %	Z_eff_	Error %
**Skin**	Phantom	3.59776E+23	0.163	7.22	0.558
	ICRU	3.60362E+23		7.2630298	
**Fibroglandular Tissue**	Phantom	3.39E+23	0.0315	7.33E+00	5.76
	ICRU	3.17967E+23		6.93E+00	
**Adipose Tissue**	Phantom	3.19937E+23	0.619	6.391046106	0.956
	ICRU	3.17967E+23		6.330516684	
**Pectoral Muscles**	Phantom	3.43E+23	0.00415	8.29E-01	0.195
	ICRU	3.44E+23		8.184042E-1	
**Carcinoma**	Phantom	3.57E+23	2.93	7.46E+00	4.46
	ICRU	3.31E+23		7.11E+00	

**Fig 9:**
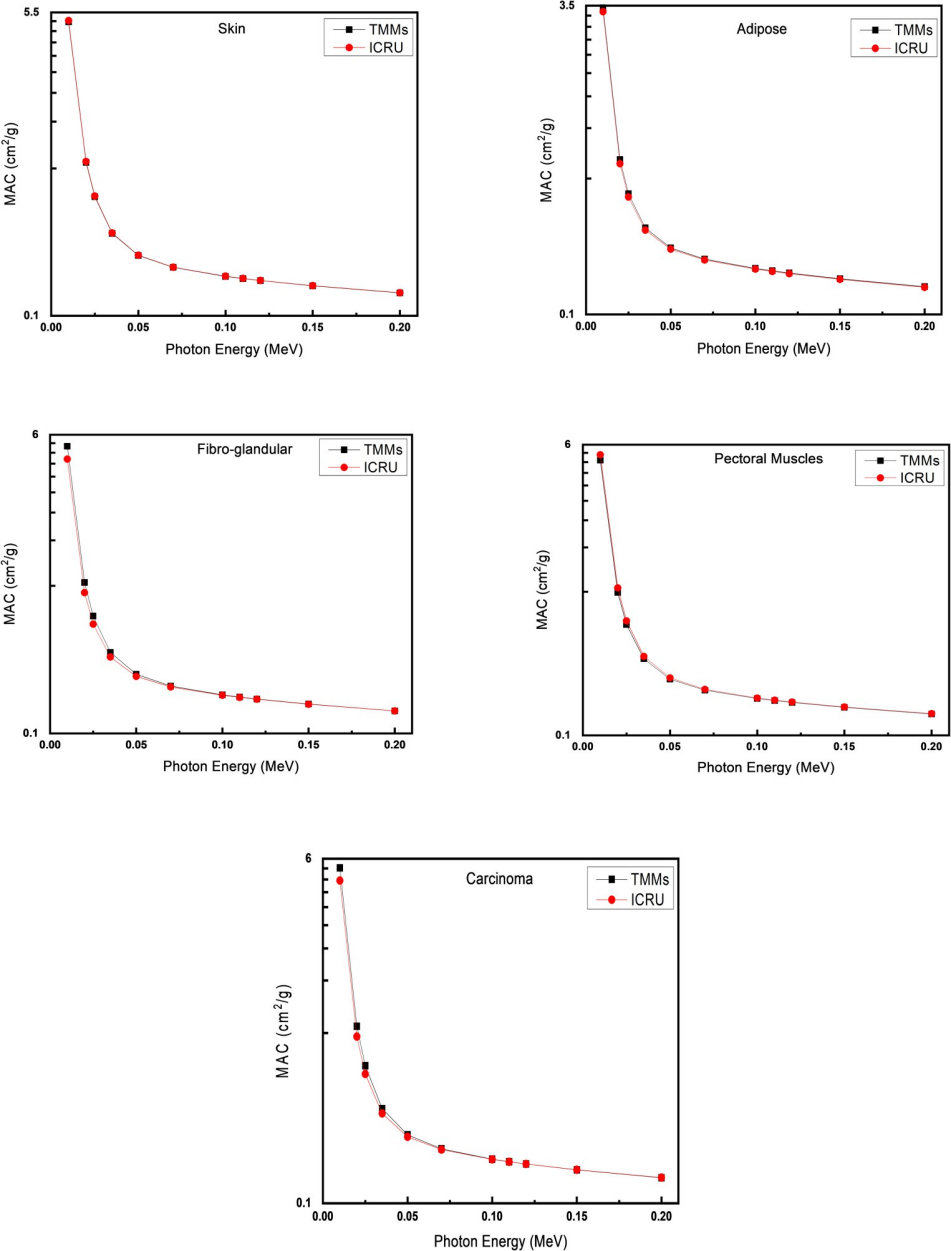
Mass attenuation coefficient versus photon energy of natural and mimicked skin, adipose, fibroglandular, pectoral muscle, and carcinoma tissue. Y-axes are in Logarithmic scales.

The MAC against the applied photonic energy for the real and mimicked skin, adipose tissue, fibroglandular tissue, malignant carcinoma, and pectoral muscle tissue are shown in Fig 9. All the tissue-mimicking materials introduced in the proposed phantom revealed a good agreement in all the energy ranges with insignificant differences in the range of small applied photonic energy. The MAC for the skin and adipose tissue natural and calculated results showed great overlapped graphs for all points with high similarity.

### 5.2. Non-ionizing properties of the chosen TMMs

For using the proposed phantom in MRI, the spin-lattice (T1) and Spin-Spin (T2) relaxation times are investigated using our preclinical 0.5 Tesla MRI. These are the intrinsic timing parameters that are inherited within the tissue, and they are responsible for the contrast of MRI images. For breast MRI, the five TMMs were characterized by measuring their T1 and T2 relaxation times. Table 4 shows the measured T1 and T2 relaxation times of the mimicked adipose, pectoral muscle, skin, fibroglandular, and carcinoma tissue, at 0.5 Tesla. An agreement has been achieved for the tissue of the adipose and muscles with the literature, indicating the possibility of observing them in MRI when T1W and T2W image contrast techniques are used. To the best of our knowledge, there are no references for the remaining natural tissue at the measured field strength.

**Table 4:** Comparison of the measured T1 and T2 relaxation times for natural and mimicked tissue at 0.5 Tesla.

Tissue	T1		T2	
	Reference at 0.5 T	Measured at 0.5 T	Ref. at 0.5 T	Measured at 0.5 T
**Adipose tissue**	0.102s [23]	0.131s	0.08s [23]	0.082s
**Pectoral muscle**	0.560s [23]	0.586s	0.034s [23]	0.053s
**Skin**	No ref.	0.172s	No ref.	0.073s
**Fibroglandular**	No ref.	0.819s	No ref.	0.051s
**Carcinoma**	No ref.	0.569s	No ref.	0.058s

### 5.3. Phantom clinical validation

The fabricated breast phantom was clinically validated at KFSH using various imaging modalities. The phantom was screened with CT and mammography as ionizing radiation machines and breast MRI as non-ionizing machine as soon as the phantom fabrication was completed. The ultrasound and photoacoustic properties of the fabricated TMMs have not been tested.

**Fig 10:**
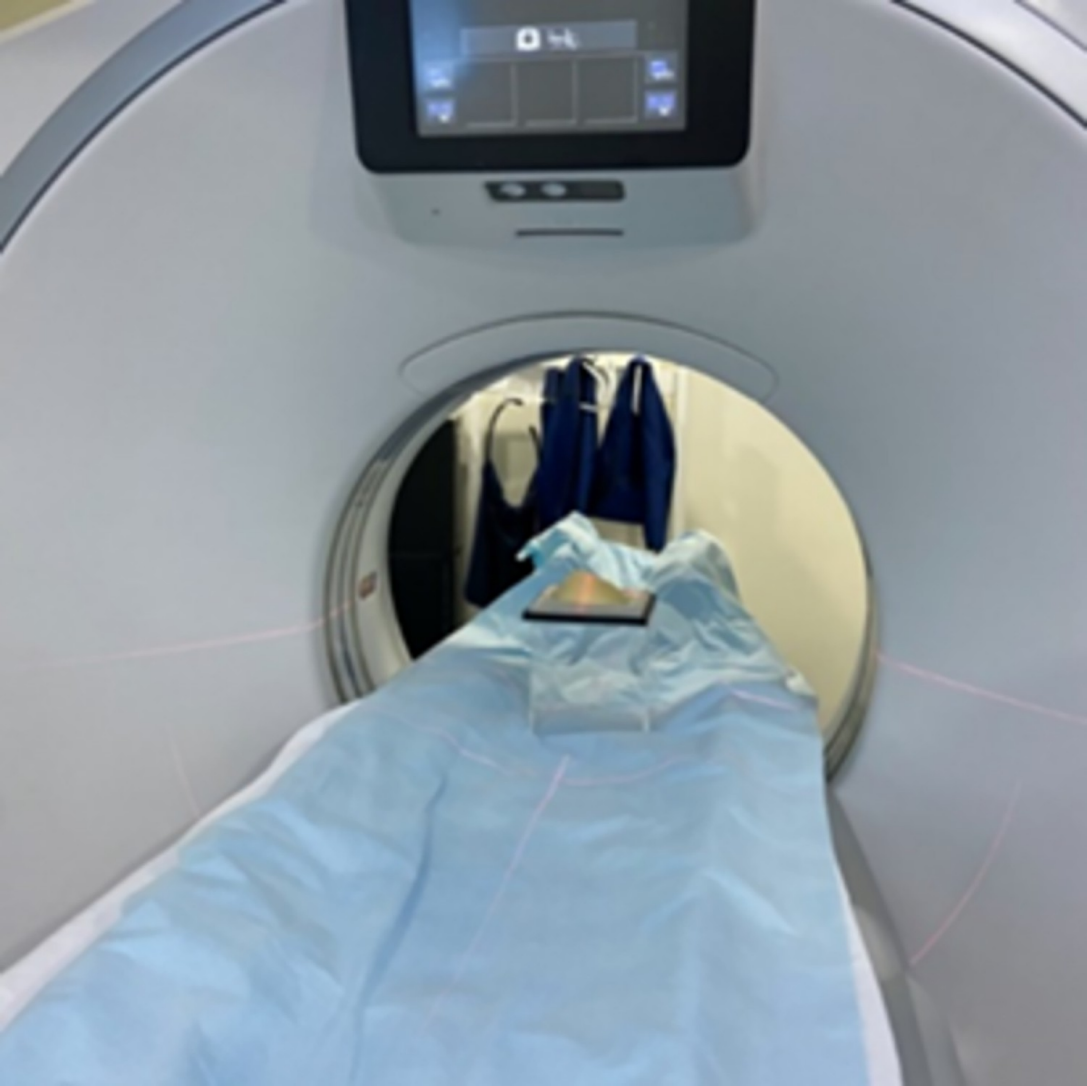
The fabricated breast phantom placed inside CT for imaging.

**Fig 11:**
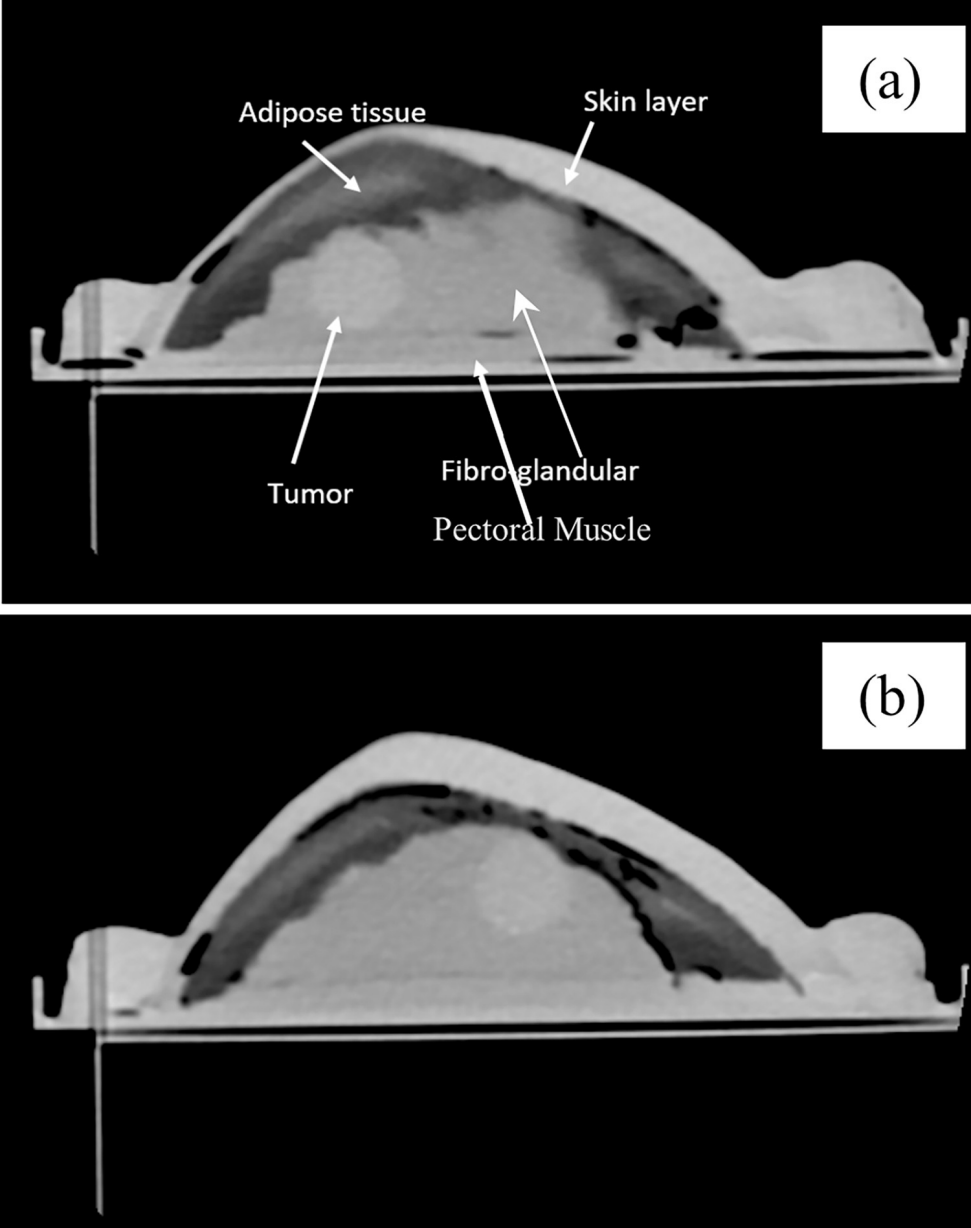
Breast Phantom CT sagittal image for the (a) left tumor and (b) right tumor.

Fig 10 shows the breast phantom before CT imaging. Several-cross sections were taken for the phantom. Fig 11(A) and 11(B) present the phantom CT sagittal images, clearly showing the left and right tumors, respectively. All fabricated tissue were able to be distinguished from each other, and the two introduced malignant tumors appeared. Inside each TMMs, the air bubbles were eliminated. Even though the process found in the literature to avoid phantom air bubbles was applied, by allowing the mixture for each tissue layer to cool down enough before pouring the followed tissue mixture; however, some air bubbles were found between the tissue layers. Going from the outer layer into the inner layer, the skin appears followed by adipose tissue, fibroglandular, carcinoma, and pectoral muscle. It is essential to mention that during skin molds fabrication, the mechanical properties such as handling the whole fabricated tissue inside it without any corrosion cannot be reached with the real skin thickness. The fabricated skin thickness has been increased for this reason. The breast scan process was taken with a real patient protocol, and images were analyzed on SYNAPSE (Fuji Film, Inc., Tokyo, Japan). When the whole tumor was taken as a region of interest (ROI), the mean CT number and the standard deviation (SD) were 87.467 HU and ±7.575 HU, respectively. The CT values achieved for primary breast cancer HU CT values are matching to [24], where the mean CE-CT HU of the primary tumor was 91.92 and the SD was ±28.36 HU. We also observed that the carcinoma HU value was consistent with the delated ducts [25]. For another region of pure fat tissue, the average CT number was -55 HU, with an SD of ±23.445 HU. The fabricated fibroglandular, pectoral muscle, and skin tissue revealed mean CT and SD HU values of 60.033±4.414, 62.367±10.334, and 132.493±4.45 HU, respectively. These values are found to be consistent with [12, 25, 26].

Table 5 shows a good match between the reference and measured HU values for most of the tissue. The measured HU values are within the range of natural tissue, and the slight difference from some references is attributed to the difference in the energy and imaging protocol used. The standard deviations in the analysis of some tissue remain congruent with the higher variances observed in the actual tissue compositions [25].

**Table 5:** The measured and reference mean and standard deviation HU values.

Tissue	Mean		StdDev	
	Measured	Reference	Measured	Reference
	CT HU	CT HU	StdDev	StdDev
**Tumor**	87.467	91.92 [24]	±7.575	±28.36 [24]
		72.5 [25]		±5 [25]
		65.94 [12]		±31.5 [12]
**Adipose tissue**	-55	-108.75 [12]	±23.445	±17.2 [12]
		-68.7 [25]		±2.7 [25]
**Fibroglandular tissue**	60.033	65.2 [25]	±4.414	±6 [25]
		46.88 [12]		± 23.9 [12]
**Pectoral muscle**	62.367	52.25 [12]	±10.334	±25.6 [12]
		−29 to 150 [26]		
**Skin**	132.493	100.75 [12]	±4.45	±28.9 [12]

**Fig 12:**
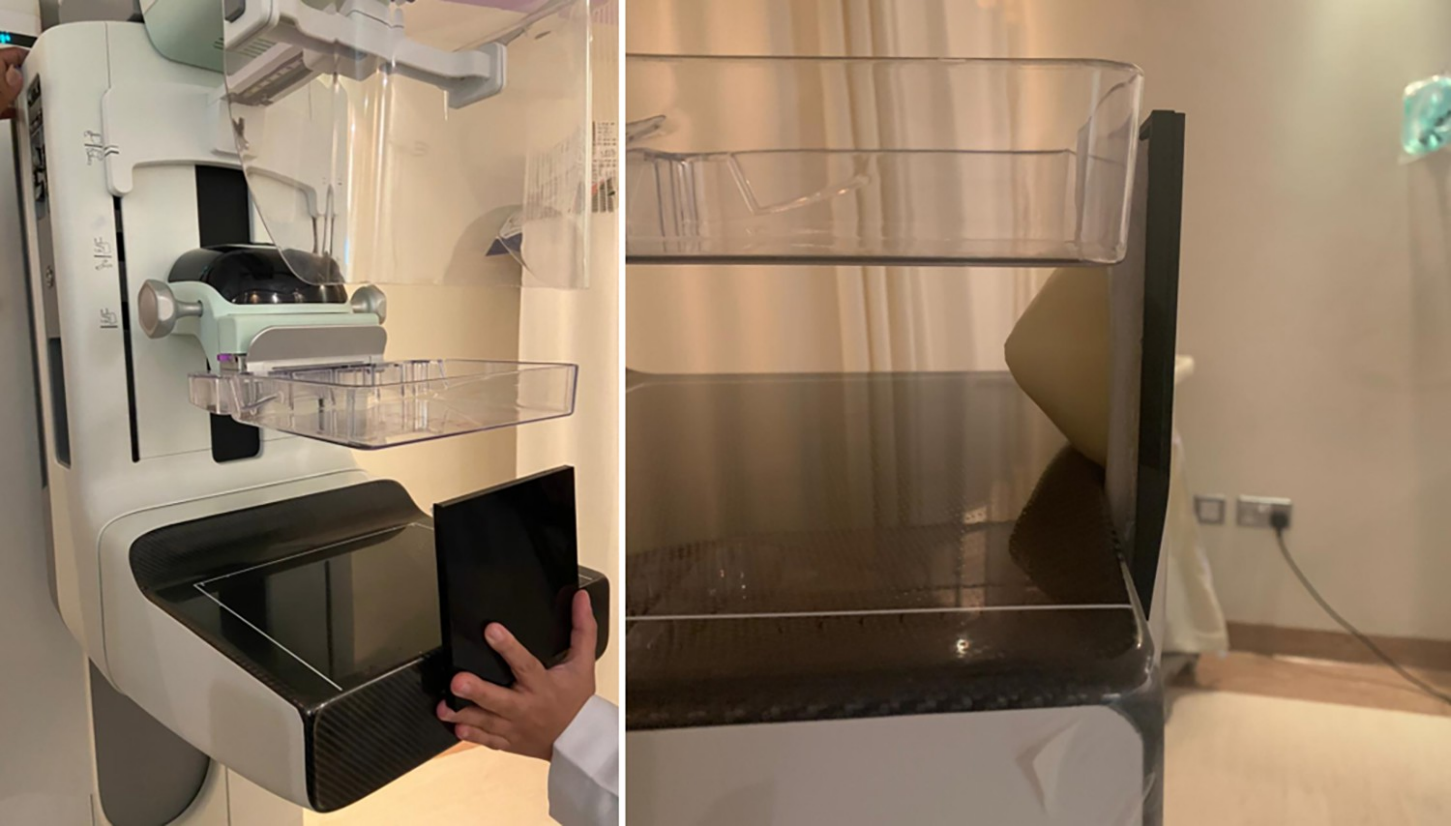
Breast phantom in mammography imaging.

**Fig 13:**
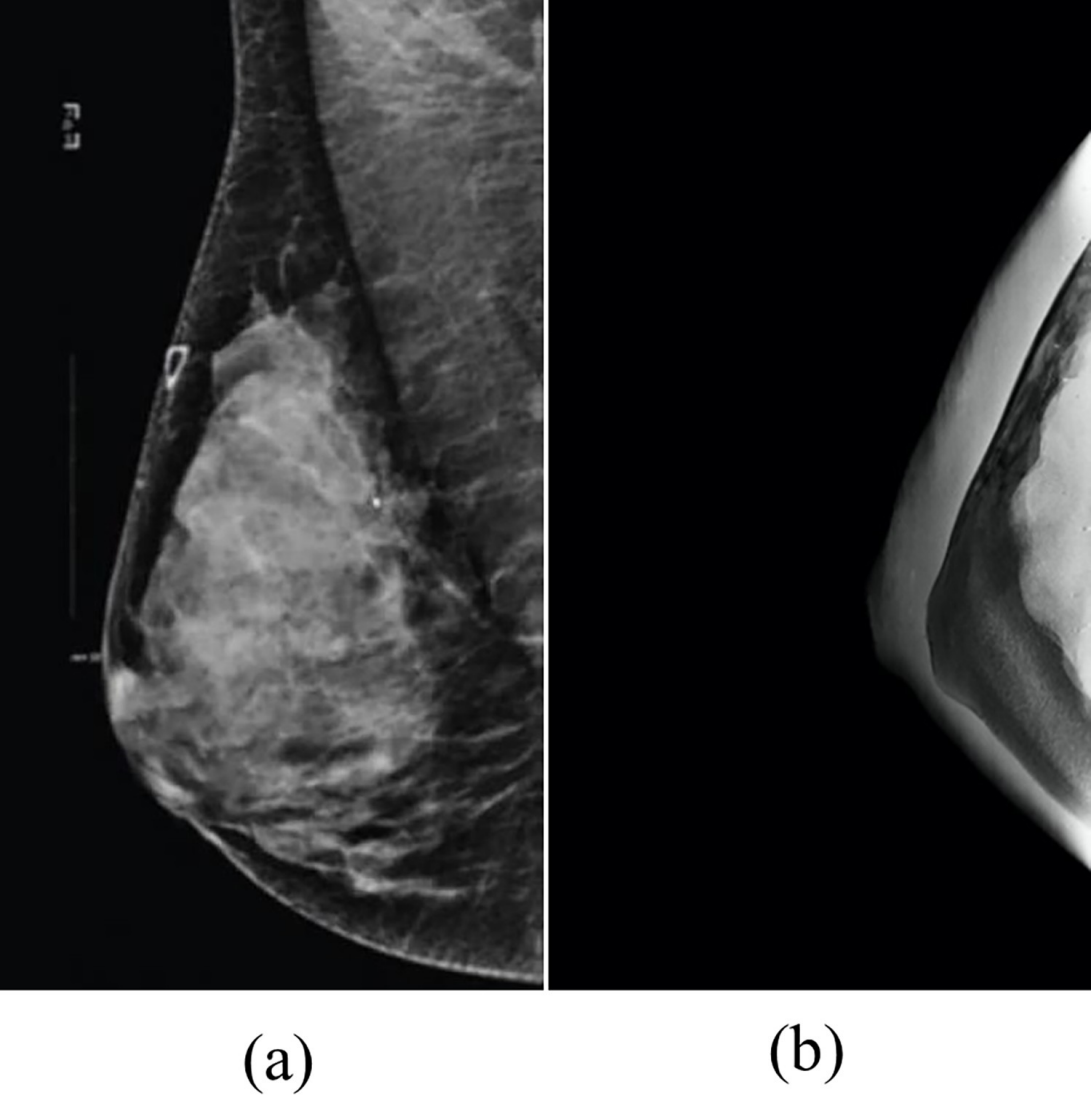
MAMO image for (a) Mediolateral oblique (MLO) view of natural breast and (b) Craniocaudal (CC) view of the fabricated phantom.

The introduced breast phantom was also screened with mammography, as shown in Fig 12. Fig 13(A) and 13(B) show two images of a natural breast and the fabricated breast phantom. Given that the phantom contains an incompressible adipose tissue, it was expected not to accurately diagnose the developed carcinoma inside the breast. However, compared to the natural breast, the phantom provided a good morphology. Further, the grayscale of each breast tissue in the phantom was approximately within the natural breast tissue grayscale range.

**Fig 14:**
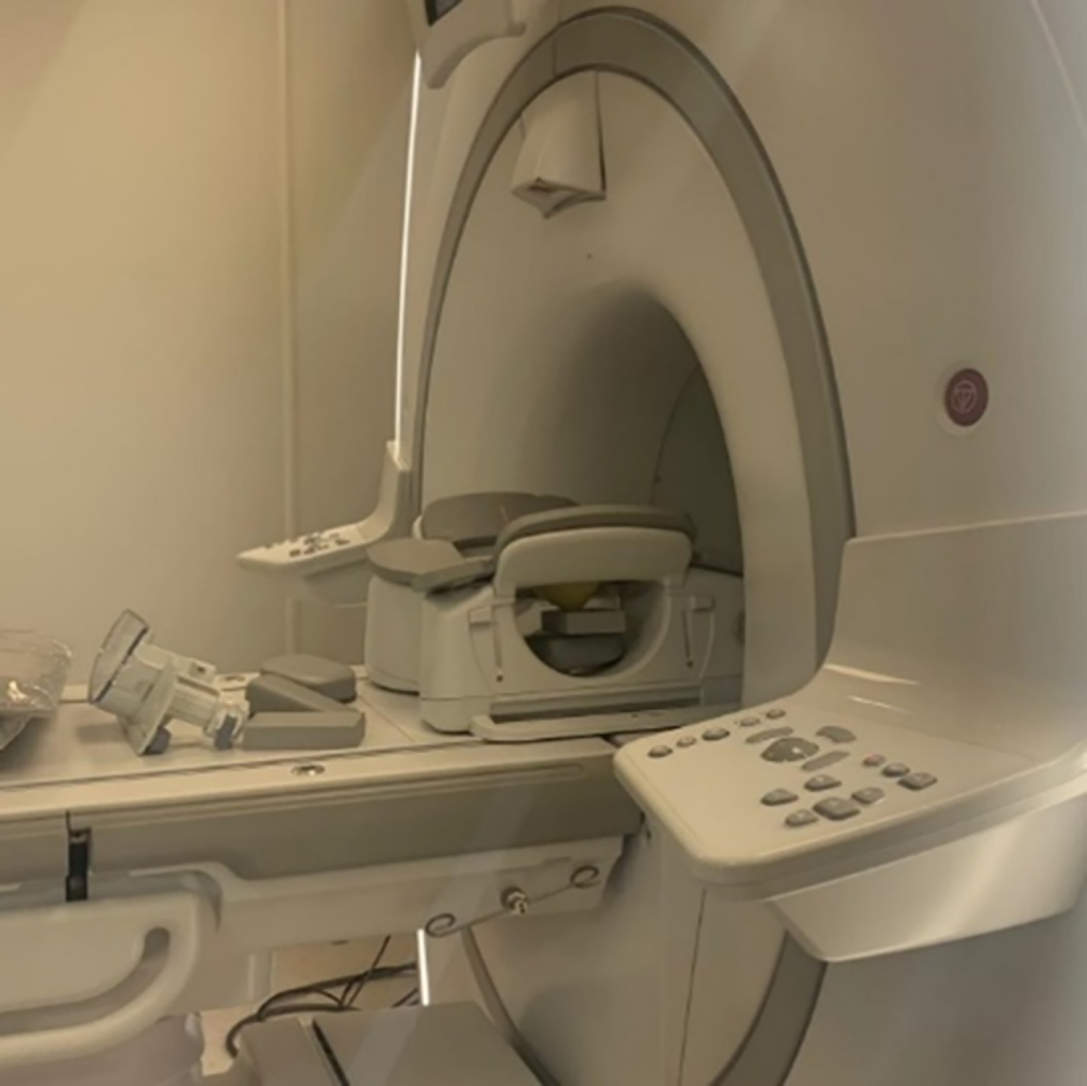
The Breast phantom setting inside the breast MRI coil.

**Fig 15:**
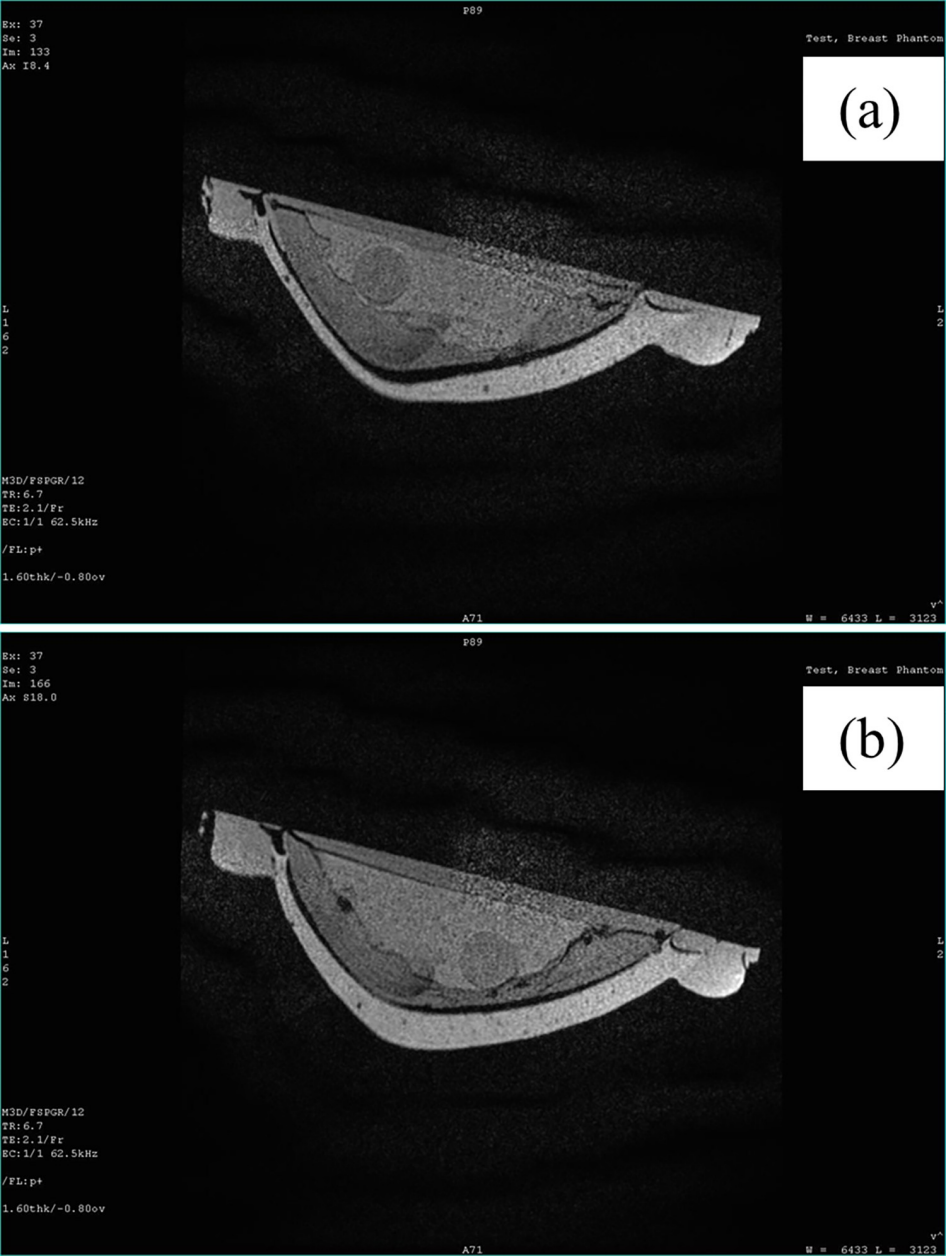
3T T1 breast phantom MRI T1 weighted image. (a) left tumor (b) right tumor.

**Fig 16:**
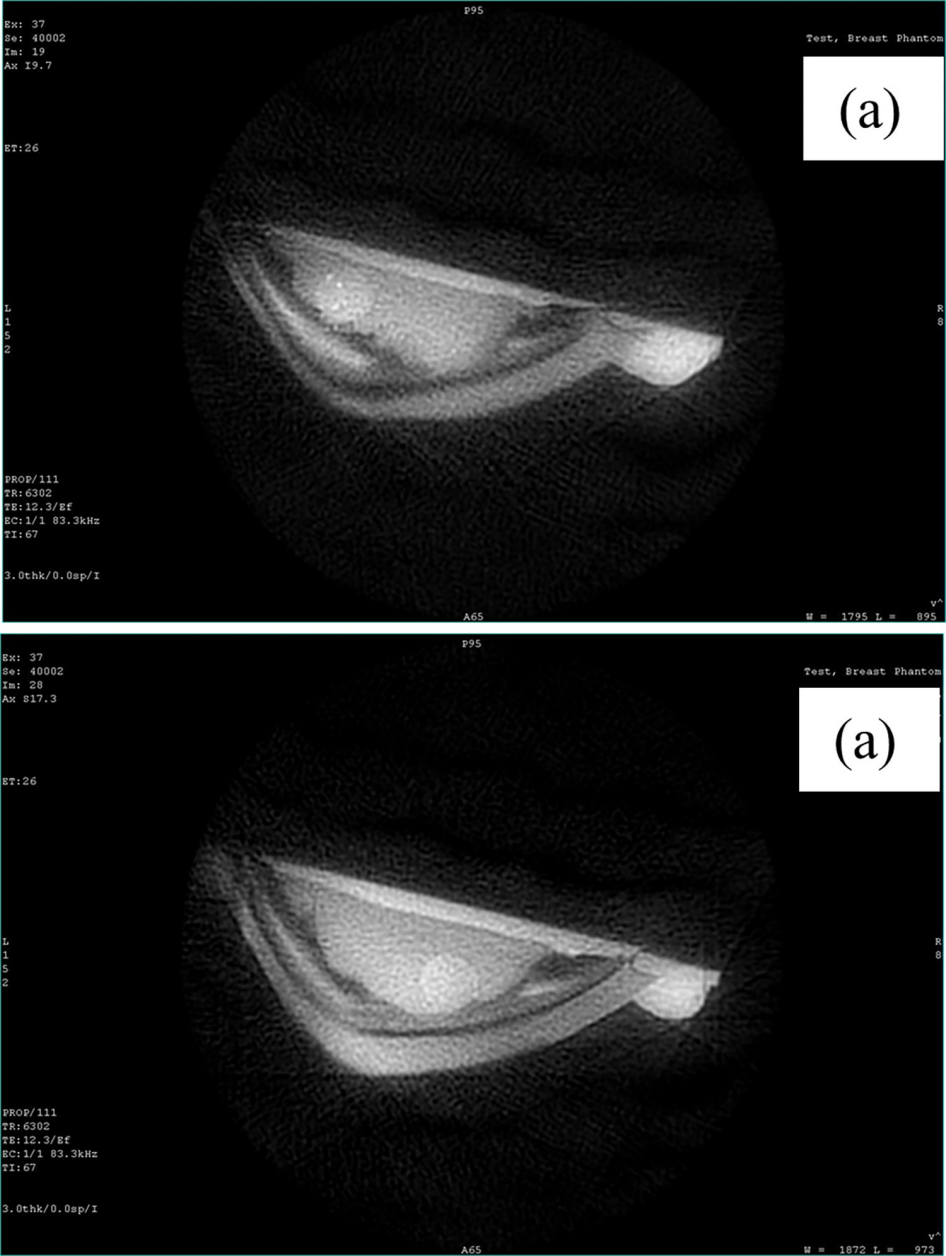
3T T2 breast phantom MRI T2 weighted image. (a) left tumor (b) right tumor.

Fig 14 shows the breast phantom for MRI imaging. The T1 weighted images were taken as shown in Fig 15(A) and 15(B) to image the phantom with the left and right tumors, respectively. Similarly, T2W images were taken as shown in Fig 16(A) and 16(B) to image the phantom with the left and right tumors, respectively. Since the tumors simulate watery malignant carcinomas, they appeared bright in T2W images, and appeared dark in T1W images. Additionally, the adipose tissue which is mainly fat appeared bright in T1W because fat quickly realigns its longitudinal magnetization with the static magnetic field B0 of the MRI magnet. The adipose tissue appeared dark during T2W.

## 6. Conclusion

In this work, we developed a patient-based heterogeneous breast phantom that mimicked skin, adipose, fibroglandular, muscle tissue, and carcinoma in the breast while screened with ionizing and non-ionizing imaging modalities. Each chosen tissue mimicking materials of the phantom was validated using three main ionizing radiation characteristics, which are the mass attenuation coefficient, electron density, and effective atomic number. On the other hand, the TMMs were characterized for non-ionizing radiation by their T1 and T2 relaxation times using a preclinical MRI unit. The process of developing the phantom started by the segmentation process using the 3D Slicer and the refinement process using the Meshmixer software. Then, the designed molds were printed using PRUSA 3D printers. Concerning the fabrication process, a combination of different materials with different quantities was tailored to match the response of the real breast tissue as possible. The weight fractions of the elements used in these materials were calculated numerically before being compared with the values from the ICRU reports. The MAC, electron density, and effective atomic number for each TMM showed a good agreement with those parameters calculated from the ICRU reference values. Furthermore, most of the relaxation times for the fabricated tissue showed a good correlation with their natural relaxation times. We performed the experimental validation of the fabricated phantom using CT, MRI, and MAMO machines. The TMMs of the fabricated phantom appeared with average CT HU values within the range of natural tissue and acceptable standard deviations. Therefore, the TMMs’ grayscale colors were the same as in real tissue. T1W and T2W images in MRI exhibited the same contrasts and colors as in fatty, watery, and other natural breast tissues. The carcinoma introduced in the phantom appeared clearly on CT and MRI. However, since the developed phantom was not compressible (which can be resolved in future work), the carcinoma could not be observed in mammography. The introduced work will provide a good reference for those working on developing 3D Voxel-based numerical breast phantom models.

## Supporting information

S1 Data (RAR)
